# Minimum 4-Year Outcomes of Cervical Total Disc Arthroplasty Versus Fusion: A Meta-Analysis Based on Prospective Randomized Controlled Trials

**DOI:** 10.1097/MD.0000000000000665

**Published:** 2015-04-17

**Authors:** Ai-Min Wu, Hui Xu, Kenneth Paul Mullinix, Hai-Ming Jin, Zhe-Yu Huang, Qing-Bo Lv, Sheng Wang, Hua-Zi Xu, Yong-Long Chi

**Affiliations:** From the Department of Spinal Surgery (A-MW, HX, H-MJ, Z-YH, Q-BL, SW, H-ZX, Y-LC), Second Affiliated Hospital of Wenzhou Medical University, Zhejiang Spinal Research Center, Wenzhou, Zhejiang, People's Republic of China; and Department of Orthopaedic Surgery (KPM), Orthopaedic Spinal Research Institute, University of Maryland St Joseph Medical Center, Towson, MD, USA.

## Abstract

The prevalence of cervical disc disease is high, and the traditional surgical method of anterior cervical discectomy and fusion (ACDF) carries with it the disadvantages of motion loss at the operated level, and accelerated adjacent level disc degeneration. Preliminary results of the efficacy and reoperative rate comparing TDA versus ACDF have been reported; however, the long-term outcomes of TDA versus ACDF still remain a topic of debate.

This review was prepared following the standard procedures set forth by the Cochrane Collaboration organization, and preferred reporting items for systematic reviews and meta-analyses (PRISMA). The only studies included were randomized controlled trials with a minimum of 4 years of follow-up data. The meta-analysis included the neck disability index (NDI), visual analog scale (VAS) of neck and arm pain, SF-36 physical component scores (SF-36 PCS), over success, neurological success, work status, implant-related complications, and secondary surgery events.

Four randomized controlled trials meet the inclusion criteria. The long-term improvement of NDI, VAS of neck and arm pain, SF-36 PCS, over success, and neurological success favored the TDA group. The TDA group also had a lower incidence of secondary surgery for both the index level (RR: 0.45 [0.28, 0.72]) and adjacent level (RR: 0.53 [0.33, 0.88]).

In this meta-analysis of 4 included RCTs with a minimum 4 years of follow-ups, total disc arthroplasty showed improvements over ACDF as measured by the NDI, VAS of neck and arm pain, and SF-36 PCS.

## INTRODUCTION

Anterior cervical discectomy and fusion (ACDF) is widely used to treat cervical disc disease.^1,2^ However, this technique is in question because of the disadvantages of motion loss at the operated level and accelerated adjacent level disc degeneration.^3,4^ For this reason, the long-term efficacy and reoperative rate comparing TDA and ACDF are still in debate. Novel dynamic techniques to preserve the segmental motion of operative levels have been investigated by surgeons, including devices such as the Bryan disc, Prestige disc, Kineflex|C, and ProDisc-C.^5–8^

The potential advantages of these dynamic devices may be that they can postpone the degeneration of adjacent level disc, and avoid secondary operations on adjacent level discs. There have been many studies^9–12^ on the efficacy and reoperative rates of total disc arthroplasty (TDA) by dynamic device versus traditional ACDF; however, the results have been confounded by the secondary effects spinal decompression has on both methods.^13,14^

Therefore, the purpose of this study was to use meta-analysis to systematically review the long-term outcomes of TDA versus ACDF for cervical disc disease.

## METHODS

This is a meta-analysis of previous reports, so ethical approval is not necessary.

Inclusion criteria: prospective randomized controlled trials (RCTs) comparing TDA with dynamic devices and standard ACDF for cervical disc disease, which included symptomatic cervical disc disease with refractory/intractable radiculopathy or myelopathy; adult population; minimum of 4 years of follow-up data.

Exclusion criteria: non-RCTs or respective studies; follow-up less than 4 years; duplicated publications from the same medical center or investigate site.

Prospective RCTs with follow-up less than 4 years, nonrandomized studies, retrospective studies, and case series are excluded.

### Search Strategy and Study Selection

Two authors (A.M.W. and N.F.T.) independently searched the electronic literature database of Medline and Embase for RCTs comparing TDA with dynamic devices and standard ACDF for cervical disc disease. All studies published between January 1966 and December 2013 were included in the search strategy, without limitation of language. Keywords were used as: TDA, cervical disc arthroplasty, total disc replacement, dynamic device, artificial disc, ACDF, cervical spine arthrodesis, RCT, controlled clinical trial, randomized, randomly, and trial and are used in combination with Boolean operators of AND, OR, and NOT. The function of “related article” is also used for search. The reference studies of previous systematic reviews, meta-analysis, and RCTs were manually searched to avoid initial miss. After 2 authors assessed the potentially eligible studies independently, any disagreement was discussed and resolved with the third independent author (Y.L.C.). A track search was performed on October 1, 2014, to include the new studies published between January and October 2014

### Data Collection

After confirming the study is eligible for inclusion, 2 authors (H.X. and A.M.W.) independently extracted data for analysis. A standard data extracted form was used at this stage, including publish date, study design, sample size, follow-up duration, characteristics of patients, interventions, NDI scores, VAS of neck pain, VAS of arm pain, SF-36, over success, neurological success, work status, implant-related complication (implant loosing, implant migration, implant subsidence) and secondary surgery of index and adjacent levels.

Secondary surgery includes revision, removals, supplemental fixation, and reoperation. According to the included studies, revision was the procedure to modify the original implant, removal was the procedure that 1 or more components of the original implant configuration were removed without replacement, supplemental fixation was the procedure of implanting additional instrumentation, and reoperation of index level was any surgical procedure at the index level that did not remove, modify, or add any components.

### Risk of Bias Assessment

We assessed the risk of bias of included RCTs according to the *Cochrane Handbook for Systematic Reviews of Interventions*: random sequence generation; allocation concealment; blinding of participants and personnel; blinding of outcome assessment; incomplete outcome data addressed; selective reporting; 7. other bias. And the judgments of reviewers are classified as “low risk,” “high risk,” or “unclear risk” of bias.

### Statistical Analysis

The data suitable for meta-analysis were performed with the STATA software (version 12.0; StataCorp, College Station, TX). Relative risk (RR) was calculated for dichotomous outcomes and weighted mean difference (WMD) was calculated for continuous outcomes in this study. Sensitivity analysis involved removing 1 study and evaluating whether the other results would be markedly affected. Heterogeneity was evaluated using the χ^2^ and I^2^. We defined the acceptable heterogeneity by *P* value of χ^2^ test >0.10 and I^2^ <50%. Results of homogeneity data were pooled using the fixed-effects model and 95% confidence intervals, and heterogeneity data by random-effects models.

## RESULTS

### Included Studies and Risk of Bias Assessment

**FIGURE 1:**
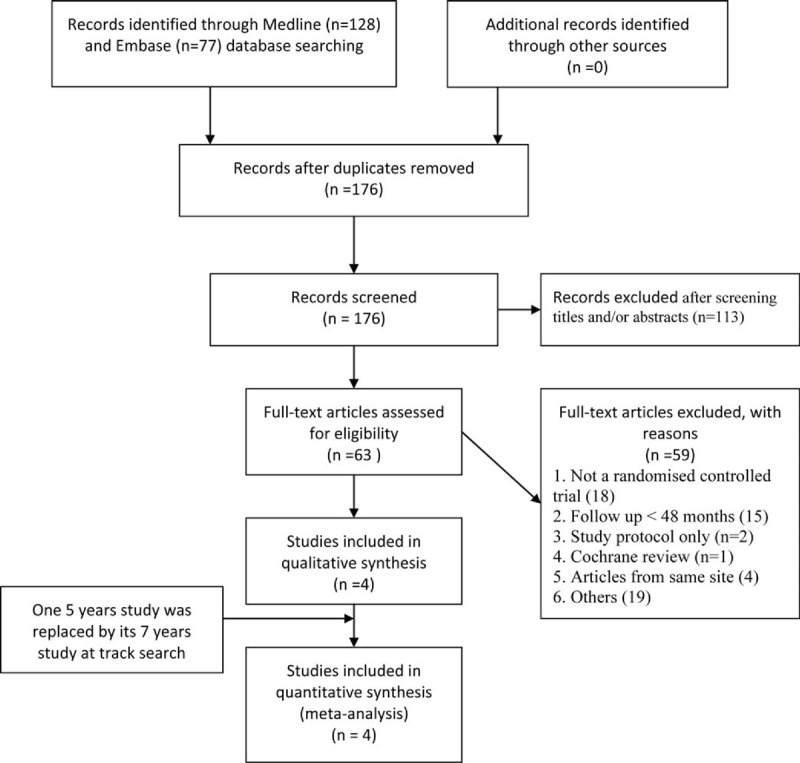
The selection of literatures for included studies.

A total of 205 records were identified through Medline (n = 128) and Embase (n = 77) databases. Twenty-nine duplicated papers were deleted, leaving 176 records. Total 63 full-text articles were assessed for eligibility, 18 literatures of them were not RCTs and 15 were minimize follow-up less than 48 months, 2 were study protocols, 4 were from the same site to the including studies, 1 was Cochrane review, and 19 for other reasons were excluded (the list of the these articles is shown in **Supplemental List 1**). At last 4 randomly controlled trials with a minimum of 4 years of follow-ups were included in this study according to our inclusion criteria, a 7-year study reported by Burkus et al^15^ was found at track search to replace their 5 years’ results^16^ (Figure 1).

The characteristics of the 4 included studies are shown in Table 1. A total of 921 patients with cervical disc disease were randomized to the TDA group (n = 506) and ACDF groups (n = 415), respectively.

**TABLE 1:**
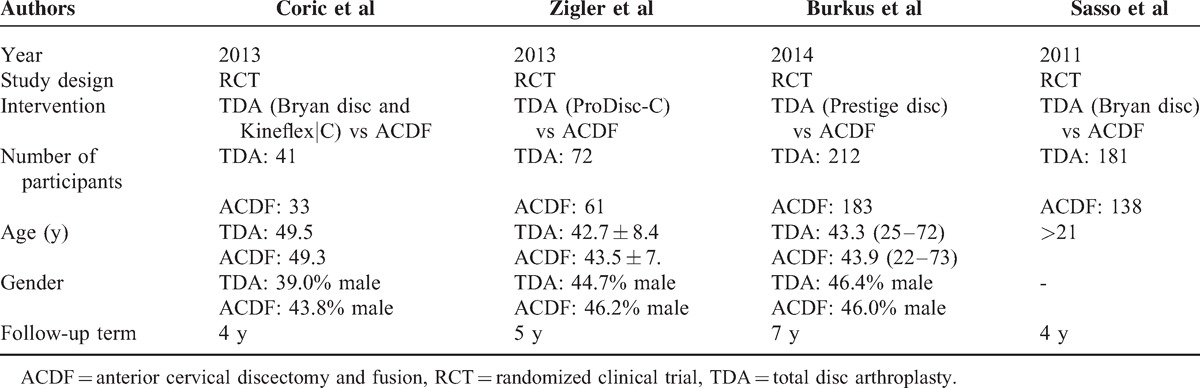
Characteristics of the Included Trials

Three included studies^15,17,18^ were multicenter randomized controlled clinical trials and 1 was from a single investigational site,^19^ the indications in study of Coric et al^19^ were 1-level symptomatic cervical disc disease with medically refractory radiculopathy, in study of Zigler et al^17^ was intractable, debilitating radiculopathy, in studies of Sasso et al^18^ and Burkus et al^15^ was 1-level cervical disc disease with radiculopathy or myelopathy, but only C3–4 or C6–7 levels included in study of Burkus et al.^15^ Information of allocation concealment is not available for all 4 studies. Two of the studies^17,18^ were blind to the participant, and 1 of the studies^15^ was not blind, 1 was unclear.^19^ Due to the difference in appearance of the postoperative radiological data between TDA and ACDF groups, a blind outcome assessment was impossible; the term “blinding of outcome assessment” was assessed as “high risk” for all 4 studies. The risk of bias of included studies is shown in Table 2.

**TABLE 2:**
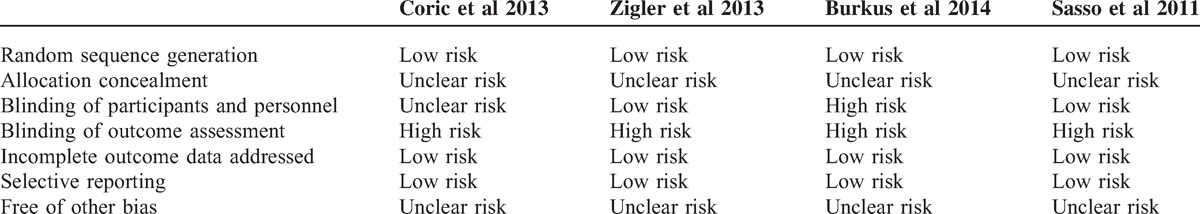
Risk of Bias Assessment of All Included Studies

### Clinical Outcomes

Two studies of Sasso et al and Burkus et al^15,18^ provide both mean value and standard deviation of the neck disability index (NDI), VAS of arm pain and neck pain, SF-36 physical component scores, over success, neurological success, and work status.

**FIGURE 2:**
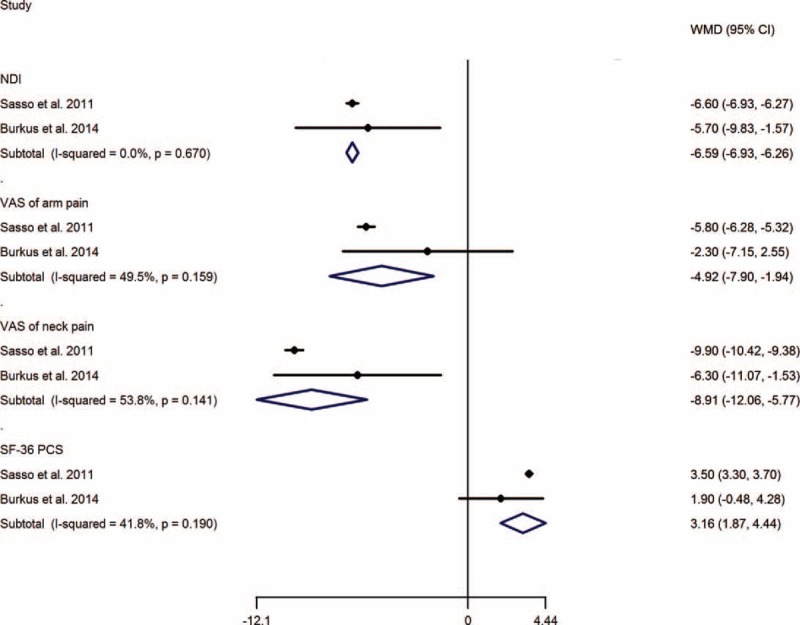
TDA versus ACDF for cervical disc disease: forest plot for NDI, VAS of arm pain and neck pain, SF-36 PCS. ACDF =  anterior cervical discectomy and fusion, CI = confidence interval, NDI =  neck disability index, SF-36 PCS =  SF-36 physical component scores, TDA =  total disc arthroplasty, VAS =  visual analog scale, WMD = weighted mean difference.

The meta-analysis of NDI, VAS of arm pain and neck pain, SF-36 physical component scores (SF-36 PCS) results showed in favor of TDA group, with RRs (95% CI) of −6.59 (−6.93, −6.26), −4.92 (−7.90, −1.94), −8.91 (−12.06, −5.77), and 3.16 (1.87, 4.44), respectively; all of them had statistical significant difference (Figure 2). Only the I^2^ of VAS of neck pain subgroup was 53.8%; the others were less than 50%.

**FIGURE 3:**
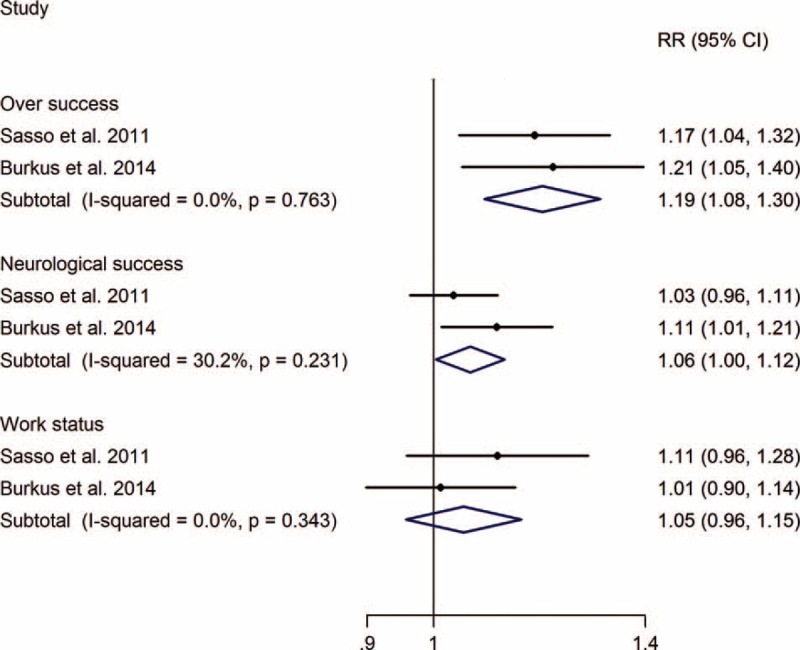
TDA versus ACDF for cervical disc disease: forest plot for over success, neurological success, and work status. ACDF =  anterior cervical discectomy and fusion, CI = confidence interval, RR =  relative risk, TDA =  total disc arthroplasty.

The results of over success and neurological success showed a higher success rate in TDA group than that in ACDF group, with RRs (95%CI) of 1.19 (1.08, 1.30), and 1.06 (1.00, 1.12), respectively. However, no significant difference was calculated of work status, with RR of 1.05 (0.96, 1.15) (Figure 3).

### Implant Complication and Secondary Surgery

**FIGURE 4:**
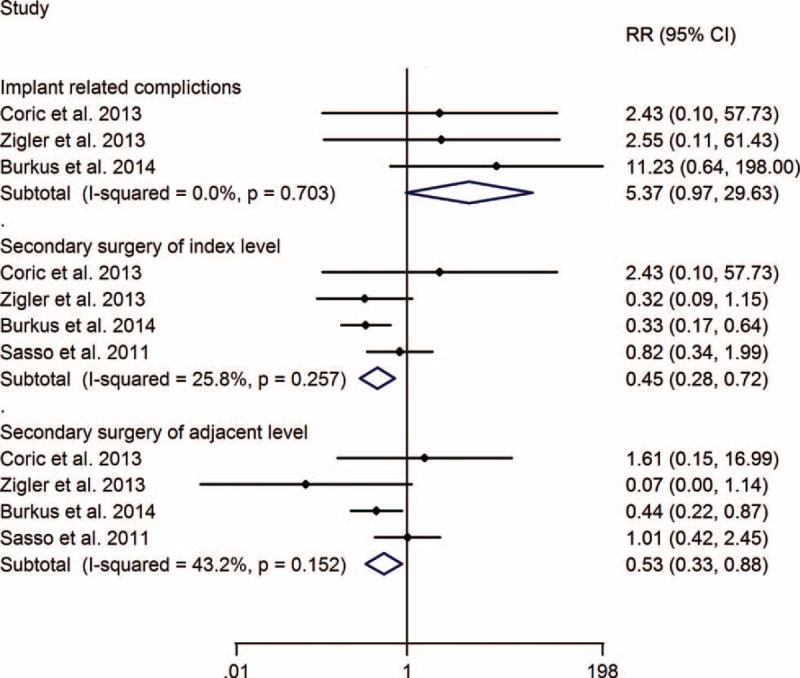
TDA versus ACDF for cervical disc disease: forest plot for implant-related complications, secondary surgery of index level, and adjacent level. ACDF =  anterior cervical discectomy and fusion, CI = confidence interval, RR =  relative risk, TDA =  total disc arthroplasty.

The RR of implant-related complications of TDA versus ACDF was 5.37 (0.97, 29.63), without significant difference, and the meta-analysis showed that TDA can significantly decrease the risk of secondary surgery at the index and adjacent levels, with RRs of 0.45 (0.28, 0.72) and 0.53 (0.33, 0.88), respectively (Figure 4).

## DISCUSSION

ACDF was one of standard treatments for cervical disc disease; many studies proved the safety and efficacy of this method.^20–22^ However, studies reporting motion loss at the operated level, and accelerated adjacent level disc degeneration, are becoming more prevalent.^11,23^ Additionally, cadaveric biomechanical studies^24,25^ suggest that significant increase in intradiscal pressure and segmental motion at the levels adjacent to fusion during normal range of motion. The drawbacks of ACDF necessitate the search for a novel device which could preserve the motion of operated levels.^26^ Although the radiological outcomes of present studies^8,11^ showed these novel dynamic devices could preserve the motion of the operative level, the ability of the device to decrease adjacent level disc degeneration was still unclear, primarily because degeneration of adjacent level discs is a slow process. In spite of these problems, there were many preliminary reports of the efficacy of dynamic devices; however, the long-term clinical outcomes and safety of motion preservation devices in the cervical spine is still in debate.

This meta-analysis including 4 prospective RCTs with a minimum of 4 years of follow-ups, 1 of them^18^ was previously included by Yang et al,^27^ Boselie et al,^28^ and Gao et al,^29^ and other 3 studies^15,17,19^ were published in 2013 and 2014, and were not included in previous systematic reviews or meta-analysis.

Our present meta-analysis showed that all the long-term improvements of NDI, VAS of neck and arm pain, SF-36 PCS, overall success, and neurological success are in favor of the TDA groups, except for work status; however, since the unclear risk of allocation concealment and high risk of the blinding of outcome assessment, these results should still be interpreted with caution.

The concept of “adjacent segment degeneration” and “adjacent segment disease” were defined differently,^30,31^ the former was defined as radiographic changes at the adjacent discs without clinical symptoms, and “adjacent segment disease” was defined as the development of new clinical symptoms based on “adjacent segment degeneration.’. For adjacent segment degeneration the patient may be symptom free, and difficult to diagnose accurately. Adjacent segment disease requiring intervention was more related to quality of life. In this study, we focused on the differences in secondary surgeries of the index and adjacent levels between TDA and ACDF groups. The long-term follow-up meta-analysis showed a lower rate of secondary surgery for the index and adjacent levels of the TDA group over ACDF groups. Although these findings were similar to the findings of Gao et al,^29^ the same reason of the unclear risk of allocation concealment and high risk of the blinding of outcome assessment, these results should still be interpreted with caution.

### Strengths of Study

Our meta-analysis compared the long-term efficacy and safety of 2 different treatment modalities, TDA and ACDF, for cervical disc disease. All of included studies were RCTs with follow-ups of more than 4 years, the longest being 7 years.^15^ Moreover, the total number of patients involved in different cervical artificial disc procedures, including, Bryan disc, Prestige disc, Kineflex|C, and ProDisc-C, was 921. Therefore, the results were credible.

### Limitations of Study

This study has several limitations. The blind to participants of 2 included studies was removed immediately after surgery; this was done to avoid the risk of bias induced by participants withdrawing before surgery; however, the risk of bias induced by further subjective evaluation is inevitable. Although the bias of random sequence generation, incomplete outcome data and selective reporting of all included studies is low risk, the blinding of outcomes assessment is high risk of all included studies, and the blinding of participants and personnel is high risk of Burkus et al,^15^ unclear risk of Coric et al,^19^ allocation concealment of all included studies is unclear risk too; therefore, the results that TDA in better clinical outcomes of NDI, VAS of neck and arm pain, SF-36 PCS, over success, and neurological success than ACDF and had a lower rate of secondary surgery in both index and adjacent levels should be interpreted with caution. Meanwhile, our present study included most patients with at least 4 years of follow-up data, but 4 studies were still a small number, and we suggest that larger sample sizes and longer-term follow-up studies could be carried out in the future.

## CONCLUSION

Our meta-analysis of 4 included RCTs with a minimum 4 years of follow-ups suggested: TDA with dynamic device was a safe and effective method for cervical disc disease, resulting in better clinical outcomes of NDI, VAS of neck and arm pain, SF-36 PCS, over success, and neurological success than ACDF, and had a lower rate of secondary surgery in both index and adjacent levels.
